# Commissioning for health improvement following the 2012 health and social care reforms in England: what has changed?

**DOI:** 10.1186/s12889-017-4122-1

**Published:** 2017-02-17

**Authors:** E. W. Gadsby, S. Peckham, A. Coleman, D. Bramwell, N. Perkins, L. M. Jenkins

**Affiliations:** 1 Centre for Health Services Studies, University of Kent, Canterbury, UK; 2 Centre for Primary Care, University of Manchester, Manchester, UK

**Keywords:** Health sector reform, Public health commissioning, Health improvement, Local government

## Abstract

**Background:**

The wide-ranging program of reforms brought about by the Health and Social Care Act (2012) in England fundamentally changed the operation of the public health system, moving responsibility for the commissioning and delivery of services from the National Health Service to locally elected councils and a new national public health agency. This paper explores the ways in which the reforms have altered public health commissioning.

**Methods:**

We conducted multi-methods research over 33 months, incorporating national surveys of Directors of Public Health and local council elected members at two time-points, and in-depth case studies in five purposively selected geographical areas.

**Results:**

Public health commissioning responsibilities have changed and become more fragmented, being split amongst a range of different organisations, most of which were newly created in 2013. There is much change in the way public health commissioning is done, in who is doing it, and in what is commissioned, since the reforms. There is wider consultation on decisions in the local council setting than in the NHS, and elected members now have a strong influence on public health prioritisation. There is more (and different) scrutiny being applied to public health contracts, and most councils have embarked on wide-ranging changes to the health improvement services they commission. Public health money is being used in different ways as councils are adapting to increasing financial constraint.

**Conclusions:**

Our findings suggest that, while some of the intended opportunities to improve population health and create a more joined-up system with clearer leadership have been achieved, fragmentation, dispersed decision-making and uncertainties regarding funding remain significant challenges. There have been profound changes in commissioning processes, with consequences for what health improvement services are ultimately commissioned. Time (and further research) will tell if any of these changes lead to improved population health outcomes and reduced health inequalities, but many of the opportunities brought about by the reforms are threatened by the continued flux in the system.

## Background

The UK government elected in 2010 embarked on a wide-ranging program of reforms to the health and social care systems in England. The Health and Social Care Act (2012) formed the centrepiece of the reforms, introducing extensive changes to the organisation, structure and delivery of health services. As part of these changes, key public health functions were transferred from the National Health Service (NHS) to local government councils. This transfer included specialist public health staff and the budget for commissioning a range of public health services, including sexual health services, public health nursing, drug and alcohol treatment, smoking cessation and weight management services. In addition, a national public health agency (Public Health England, PHE) was established, as the national leadership body for public health to provide national campaigns and co-ordinate health protection, and as an active partner in local initiatives where appropriate [1].

During the reforms, the government highlighted a number of issues that lay behind inadequate population health outcomes. It felt the system was fragmented, lacked integration and synergies across services and had overlapping responsibilities [2, 3]. It also felt the system disempowered public health professionals, insufficiently valuing their skills [3]. Crucially, the government argued that there was an insufficient focus on the root causes of ill health, and pointed to a lack of accountability with regards to outcomes. Issues faced in England chimed with cross-cutting themes that emerged from a review of public health in Europe, notably: the importance of inter-sectoral working, the existence of wide inequalities between and within countries in Europe, and the knowledge gaps around what public health policies and interventions are being implemented where, and which are most effective [4].

In 2012, a new European health policy framework was developed, to support action across government and society to improve the health and well-being of populations, reduce health inequalities, strengthen public health and ensure people-centred health systems [5]. It was in this context that the UK government set out, in the English reforms, to clarify responsibilities and accountabilities, empower people and communities, and focus on the evidence of what works. They wanted a greater emphasis, at all levels, on disease prevention, and a more joined up approach, with clearer leadership. In addition, the need to achieve better results with less money was an undercurrent to the entire health and social care reforms, driven by the government’s aim to reduce their budget deficit.

Prior to the reforms in England, Primary Care Trusts (PCTs) were the NHS bodies responsible for commissioning – strategic planning and purchasing - most health services, including for public health [6]. Until 2011, PCTs also directly managed the vast majority of NHS community health services, such as district nursing, health visiting and children’s services. In 2013, PCTs were abolished and replaced by a new NHS commissioning architecture, locally led by Clinical Commissioning Groups (CCGs), and nationally led by a new independent NHS commissioning board (NHS England) [7].

Within PCTs, public health specialists tended to provide a lead role in developing strategies for meeting local health needs, and specialist clinical and public health advice to inform PCT commissioning. Whilst public health was (and remains) an inter-organisational function, with much close working between PCTs and local councils, funding remained predominantly from NHS sources, with most decisions about services and expenditure taken within an executive decision-making framework by Directors of Public Health supported by PCT Boards [6, 8].

Public health services are now funded by a public health budget, separate from the budget managed through NHS England for healthcare. This budget is decided by the Department of Health (DoH), and managed by PHE. PHE funds public health activity either through allocations to upper-tier and unitary councils, by commissioning services via NHS England, or by commissioning or providing services itself. Most locally delivered public health activities are now commissioned or provided by local councils. The structure of local government in England is complex: there are 27 areas where services are split between upper-tier county councils (taking responsibility for social care, education, transportation and strategic planning), and smaller district councils (covering e.g. housing, leisure, environmental health and planning), and there are 125 unitary councils that provide the full range of services. All of these councils are run by elected councillors, usually affiliated to a political party, who represent and engage their local population, make key decisions, contribute to policy/strategy review and development, and conduct overview and scrutiny roles. Councils have the freedom to innovate and to make changes locally, under the ‘general power of competence’, introduced by the Localism Act 2011 [9]. There are important differences, then, in the context in which local public health commissioning is now done.

The Health and Social Care Act also introduced Health and Wellbeing Boards (HWBs) as statutory sub-committees of local councils. These boards were intended to bring together the key NHS, public health and social care leaders in each local council area to work together to co-ordinate commissioning of their services. They are thus an important part of the new health commissioning landscape [10].

A House of Commons Health Committee inquiry on public health post-2013, launched October 2015, is starting to raise some important issues related to the structures, organisation, funding and delivery of public health following the reforms [11]. However, to date, little academic attention has been paid to the impact of the reforms on public health commissioning in England. This article examines key changes to the public health system following the reforms, and explores the broad function of commissioning for health improvement within the new system. It highlights some important changes in the way public health commissioning is now undertaken, in who is doing it, and in what is commissioned. It draws on findings from a 3-year research study funded by the DoH, which examined the impact of structural changes on the functioning of the public health system, and on the approaches taken to improving the public’s health. The article critically examines these findings in the context of the intentions of the reforms to create a more joined-up system with clearer leadership and greater opportunities to improve population health.

## Methods

The PHOENIX study was a 3-year research project to examine the impact of structural reforms on the functioning of the public health system in England. It was an exploratory study that took place from the time of transition (April 2013), and so could explore the ways in which the planning, organisation, commissioning and delivery of health improvement services were changing over time as the new structures bedded in. One of its objectives was to examine approaches taken to commissioning within the new system, using obesity as a focal topic.

The study incorporated multiple methods. In a scoping review [12], we analysed policy documents and responses to the reforms from key stakeholders [13], developed a picture of how the new structures were developing, and collated demographic and other data on all 152 upper-tier and unitary local councils in England. This review identified the key themes to follow up on in the next phase of the research. It also enabled the purposive selection of local councils for later case study research, conducted from March 2014 to September 2015, in five areas. The areas were purposively selected for maximum variation across a a range of characteristics related to the councils and the populations they serve (including council type, size, urban or rural location, varied socio-demographic and economic circumstances, obesity prevalence and different political control) in order to provide a diverse range of cases. The five areas (described in Table 1) encompassed 13 different councils, including unitary, upper-tier and a sample of lower-tier (district) councils, some of which had a variety of different sharing arrangements. This enabled an examination of multiple perspectives and inter- and intra-organisational relationships.

**Table 1:** Case study sites

Site	Description	Number of interviews
A	Large county council (Conservative), including sample of 2 different sized district councils and adjacent unitary council	23
B	Cluster of three urban unitary councils (two Conservative, 1 Labour) with shared DPH	13
C	Urban metropolitan unitary council (Labour)	23
D	County council (Conservative), including sample of 2 different sized district councils, adjacent county council and unitary city council	22
E	Urban metropolitan unitary council (Labour), working with network of other urban unitary authorities	22

Within the case study areas, 103 semi-structured interviews were conducted (see Table 1) with 36 council public health staff; 18 elected members; 25 council non-public health staff; 13 provider organisation staff; six CCG staff and three other staff at regional levels. Three members of the research team were allocated across the case study sites to enable each researcher to develop a deep understanding of and good relationships within each area. Fifteen meetings were observed and documentary evidence was collated to enrich our understanding of the case study areas. A further five interviews were conducted with key informants outside of the case study areas, particularly to explore national and regional level issues and relationships with/within PHE.

In the autumn of 2015, a web-based questionnaire was sent to all Directors of Public Health (DsPH), and to councillors in all 152 upper-tier and unitary councils who had a public health brief. Usable responses were received from 49% of DsPH and 32% of elected members. The questionnaire was broadly a repeat of a survey conducted the previous year (not reported in this paper). The distribution of responses from local councils was highly representative overall. Data was analysed using SPSS.

Qualitative data was analysed on a case and theme-based approach, using NVIVO 10. Multi-investigator, multi-site and multi-method triangulation was used in an ongoing and iterative process of bringing together and interrogating the data. Reflexive, narrative accounts of each case study area were shared with the research team, which was made up of experts in public health, local government, ethnography, and public policy. Rich interpretations of emergent themes across the cases were developed collaboratively, paying particular attention to roles and relationships, power/autonomy, and decision-making processes. Analysis drew on a number of integrative theoretical frameworks, employing concepts and ideas drawn from a number of different paradigms [14, 15]. Ongoing analysis of the data allowed shifts in focus according to the interplay between theory, concepts and data, enabling sensitivity to the constantly changing field of study.

Ethical approval was granted by the university research ethics committee, and research governance approvals were obtained for each case study site.

## Results

Throughout analysis, commissioning was considered as one of the broad aspects of public health activity. As a theme, it included identifying needs, reviewing service provision, deciding priorities, procuring services, and managing performance. Our research set out to examine the context for commissioning, the people/organisations involved in commissioning activities, the processes involved, and any evidence of things changing.

### The context for commissioning

The transfer of public health staff and resources into local councils from PCTs was far from straightforward, and often accompanied other system reorganisations. For instance, in one of our case study areas, staff in a PCT were separated into a council public health team, one of three CCGs, or into a provider trust. One children’s public health commissioner who was formerly in the children’s joint commissioning team in the PCT with commissioning responsibilities for the whole of the 0–19 pathway, was transferred to a council team. Her former commissioning responsibilities were split amongst different organisations, and she was now responsible only for certain elements of the healthy child programme. She explained the resulting confusion:

*“… It has caused fragmentation of the system and certainly for the 0–19 pathway or services for children, you know, the health services for children. It has meant that different parts of the system are now responsible for commissioning different elements of it …, which is challenging”* (senior public health commissioner, council, site B).

This also had implications for the sharing of information between health and council commissioners, which this officer described as being “much more difficult for us now”.

Some public health staff chose to join PHE or NHS England, and some became part of new commissioning support organisations. There was much confusion over where staff should be transferred to (sometimes depending on the proportion of their time spent on service commissioning versus service provision), and around the organisation of budgets. There were instances where this tested relationships between councils and CCGs.

Local councils received their public health staff, resources and duties at a time of unprecedented cuts to their budgets [16]. These cuts precipitated ongoing restructures within councils which sought to streamline their organisations and reduce staffing costs. The positioning of public health teams within councils varied. Our survey found that 26% (*N* = 73) of the public health teams were distinct public health directorates; 52% were sections of another directorate; and 22% had other arrangements, including merged, distributed and mixed models. DsPH also had different levels of access to key council decision-making bodies (53% of DsPH respondents were members of the council’s most senior corporate management team), and different line-management structures (47% said that they were managerially responsible to the council’s chief executive; 53% were managed by a range of other directorate heads). Consequently, DsPH were not always in the best place for strategic influence in the council.

### Commissioning processes and people involved

Decision-making within councils was found to be very different to that within PCTs. Decisions about how to spend money were subject to a greater range of decision makers and wider consultation, both across the council and amongst the public, than before. Elected members are the key decision makers within councils; the role of officers, including those in public health, is to support them. Elected members, therefore, were influencing the priorities and actions of the public health team, sometimes overtly and sometimes more subtly. 92% of elected members responding to our survey (*N* = 38) said they felt always able (45%) or quite often able (47%) to influence the priorities of the public health team. In our case studies, we saw how this influence might operate more subtly, perhaps according to the ideologies and interests of the elected member, or the politics of the council. For instance, in one Conservative-led council, the elected member explained that he would have a very difficult job persuading his cabinet to significantly increase spending on smoking cessation: *“They’re not particularly interested in it, they think … ‘oh well if people smoke themselves silly, let them smoke themselves silly’”* (elected member, council, site A).

Compared with the NHS, local councils take different approaches to prioritisation and commissioning, influenced in part by over 15 years of implementing ‘Best Value’^1^. The processes of commissioning (and new procurement laws) within a council have had to be learned by incoming public health staff. At the same time, public health staff have tried to educate councillors in public health commissioning.

Several commissioning officers who had worked within councils prior to the reforms (e.g. in adult or children’s social care directorates) and who moved, following the reforms, into the public health teams, talked about differences they observed in how commissioning was done. One, referring to her incoming public health colleagues, explained:

*“We were faced with a lot of ignorance about commissioning - local authority style commissioning and business processes - amongst our colleagues… I was shocked actually by the lack of understanding of what we had been doing or what we did [as local authority commissioners]”* (commissioner, council, site B)*.*

Another talked about the differences between commissioning in PCTs and commissioning in the councils. She explained that *“public health has commissioning responsibilities now in a way that they didn’t in the old PCT”.* She described commissioning in the former PCTs as comparatively less ‘robust’, with less accountability, and less scrutiny of performance and outcomes data:

“*there’s much stronger scrutiny in local government and that’s all areas of business and it’s something that we’ve had to really work with our providers in NHS specifically around understanding”* (commissioner, council, site A)*.*

From the point of view of providers, however, the sometimes rather narrow outcomes-based scrutiny that services were now subjected to was not always appropriate for complex public health interventions. For instance, the provider of a range of obesity prevention services in one of our case study areas complained that the focus on outcomes in terms of body mass index reductions belied the fact that most of their time and resources were spent on engaging communities and developing relationships with schools and others. The outcomes of this type of activity, however, are impossible to measure.

Having a distinct public health grant for the first time enabled DsPH to take a different approach – a more strategic approach - to the allocation of the public health budget. A public health officer in one of our sites described how, in the PCT, they were sometimes left ‘scrabbling’ around for funds, when public health priorities and PCT priorities were not always well matched. However, with a ring-fenced budget, they were able to plan how best to match spending against their local priorities. The leader of a council in site A explained how they were prepared to completely shake up the way in which the public health grant was spent: “*We’ve got to start at reviewing; is that delivering to the right priorities or not? Is it value for money or not? And what should we stop doing and what should we start doing?*” Indeed, this process of whole-scale service reviews for specific areas (such as obesity) was demanded by councillors in all of our case study areas. For public health officers, this sometimes gave them the freedom to pursue quite different approaches.

Decision-making across the local system following the reforms was intended to be more co-ordinated. However, with commissioning responsibilities now fragmented between NHS England, PHE, local councils and CCGs, our research found that co-ordination was proving to be difficult. Moreover, the lack of clarity about responsibilities sometimes led to delays in the commissioning of services, and/or tensions in the relationships between organisations. Commissioning across an obesity pathway, for instance, involves councils (for broad obesity prevention and non-intensive weight management services), CCGs (for specialist obesity services) and NHS England (bariatric services) [17, 18]. Across England, we know that there are significant gaps in this pathway, with a particular lack of specialist obesity services [19, 20]. Following the reforms, there was a great deal of confusion about whose responsibility it was to commission these services.

It is clear that, as with many public health interventions, if weight management and obesity prevention services are to achieve their objectives, primary and community care providers play a vital role. The presence, absence, type and success of health improvement services commissioned by councils have important implications for NHS work. However, there is now a greater disconnect between public health officers and NHS commissioners. In response to our survey, 48% of DsPH (*N* = 69) said they felt ‘less able’ to influence local CCGs than before the reforms. In our case study sites, we found that evidence of meaningful engagement between public health teams and CCGs was limited. This HWB chair felt that CCGs had become disengaged from public health:

“*I think we’ve got to persuade the CCG that, in particular, public health is everybody’s business, it’s not just the local authority’s business. … they see public health as a separate entity at the moment, and not part of an integrated health economy”* (Chair HWB, council, site C)*.*

HWBs were meant to be the mechanism for co-ordinating commissioning across NHS, social care and public health at the strategic level. Our survey found that amongst DsPH (*N* = 65), 48% felt the HWB was ‘definitely’ instrumental in identifying the main health and wellbeing priorities, and 45% felt it had ‘definitely’ strengthened relationships between commissioning organisations. However, less than 5% felt that the HWB was ‘definitely’ making difficult decisions, and only 28% felt that it had ‘definitely’ begun to address the wider determinants of health. A further complication with co-ordinating across the system and addressing wider determinants is that in two-tier councils, many of the functions that public health are expected to work across are based in multiple lower-tier district councils. Public health officers must therefore build relationships with a greater number of different organisations, all with their own priorities and ideas. In addition, these district councils often have a limited voice on HWBs. It is perhaps partly for this reason that some HWBs were not seen to be significantly engaging with the public health agenda. As this HWB chair explained:

*“We have a very strong focus on integration, Better Care Fund – all that side of things. I’m conscious sometimes of an element of criticism … there’s always a challenge to say, ‘Are you actually thinking enough about long term determinants and all the sort of public health agenda’ …”* (Chair HWB, council, site A).

### What has changed?

**Fig. 1:**
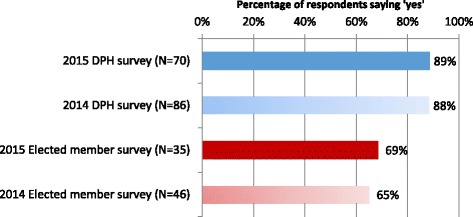
Use of public health budget to invest in other council departments in previous 12 months

Our research suggested that, as a result of the reforms, public health commissioning was changing on a number of levels. Firstly, money was being used in different ways. One indication of this was the way in which the ring-fenced public health budget was being used to invest in other departments in the majority of councils (see Fig. 1). Given the huge cuts councils were having to make, most DsPH felt that, now the public health budget was contained within the council, it was expected to contribute to the overall savings they needed to make. Many seemed reconciled that the budget would now be used to fund other services – in many cases, services that would have been cut (e.g. children’s centres) had public health funding not been available. And in our case studies, public health officers talked about the opportunities this sometimes presented, in terms of embedding public health activities and objectives within other council services and providing more joined-up ways of thinking and working.

**Fig. 2:**
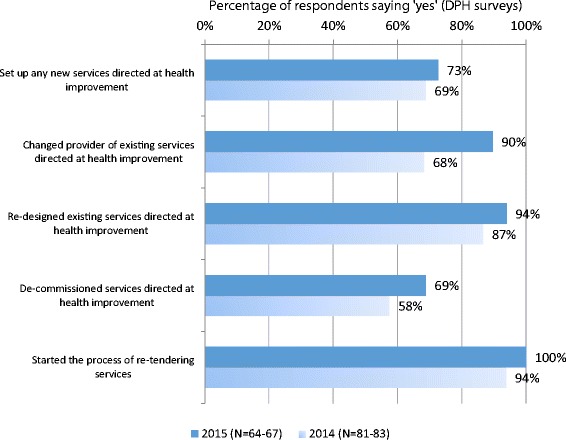
Changes made by councils to services commissioned under public health budget in last 12 months

Secondly, there were many changes being made to the commissioning of health improvement services (see Fig. 2). The move to local government prompted public health commissioners to look at services and contracts anew. In addition, councils tended towards shorter contracts and more frequent retendering of services than the NHS. All our respondents had started the process of retendering within 2 years. But we also saw the majority of responding authorities (*N* = 64–67) having set up new services (73%), changed provider of existing services (90%), re-designed existing services (94%) and de-commissioned services (69%). In our case study areas we saw that extensive commissioning changes were sometimes occurring as a result of changes in local area arrangements, for instance, where several areas (former PCTs) were brought together into one (council). Other commissioning changes, however, were as a result of service reviews that were very critical of service outcomes.

Our surveys asked for more information about changes that were being made to obesity commissioning. DsPH commented that they were wanting to move away from ineffective schemes, increase their focus on children, use new providers and create a more integrated pathway. All these changes were resulting in insecurity in the provider landscape.

Finally, there were changes to the size and profile of the public health teams responsible for commissioning health improvement services. DsPH were asked whether there had been changes in the last 12 months to the size and composition of their public health team. 28% (*N* = 72) reported that they had fewer public health specialists. 15% reported they had more business managers/commissioning support staff, and 22% (*N* = 54) said they had more ‘other’ staff (not falling into the DPH, specialist, analyst or commissioning support categories). In our case study sites, public health officers talked of the need to address skill gaps within their team in response to working in the new environment. In one of our sites, for instance, the public health commissioning team (made up of non-public health specialists) had been significantly bolstered. The team of public health specialists had been correspondingly reduced.

It was not easy to tell, at this juncture, whether these observed changes in commissioning had resulted in a significantly different set of activities being commissioned. However, there were early signs of some general shifts occurring. In three of our case study areas, we observed a shift towards the commissioning of more holistic ‘healthy lifestyle’ services, bringing together weight management, smoking cessation, alcohol reduction, sexual health services, and so on. In two of our councils, we saw a shift (at least in rhetoric) towards ‘whole council’ approaches, for instance, where they were seeking to address a broader range of factors influencing obesity, particularly by working across council departments. We witnessed a greater recognition of public health objectives and expected outcomes in a wider range of council services as a result of public health investment. And we saw public health staff working hard to influence the wider workforce. Particularly during the transition phase, as public health were settling into their new homes, a number of programmes including learning events, information sharing, and engagement events were targeted at elected members and non-public health officers across the council.

## Discussion

The reforms expressed a clear intention to simplify and streamline a previously complex, fragmented system. The transfer of public health responsibilities into local councils was to ensure that public health outcomes were embedded across a council’s functions. The creation of HWBs was to ensure strategic direction across organisations.

The functions of the now extinct PCTs were spread across CCGs, councils and provider organisations, creating a more complex organisational picture than before the reforms, with more complex accountability and governance structures. Moreover, there was continued upheaval in the system, with elements such as CCGs and public health teams merging, the PHE regional tier ‘downsizing’, and local councils constantly restructuring as they tried to cope with substantial budget cuts. Fragmentation is a problem common to many health systems, and is a condition related to the tendency within health care planning to focus and act on the parts without adequately appreciating their relation to the evolving whole [21]. There is a constant challenge to create a system focused on relationships across the whole – whole people, whole systems, whole communities. It is often these relationships across the whole that suffer in the context of financial restraint and continual change [22].

The move of public health into local councils in England created a new working environment for commissioners, public health practitioners and providers. Our findings have demonstrated how, in this new environment, existing public health capacity has been both freed and stifled. Public health professionals have the opportunity to take on a more significant role in shaping local places, but will need to find a balance between ‘service’ public health and academic ‘social medicine’ [23].

The considerable literature on decentralisation suggests that the transfer of authority and resources to local government might offer significant opportunities to improve access to health and other care services, to provide services that are better aligned to needs and local preferences, and to allow for increased flexibility and transparency [24–26]. However, the reforms in England simply moved public health responsibilities at the local level from executive decision-making bodies (PCTs) to democratically governed councils. Whilst PCTs had the same ‘local’ focus as councils, they were historically more directly accountable to central government, and, with a few exceptions, were poor at developing local ‘bottom-up’ methods for making NHS services more user-responsive [27]. Councils, on the other hand, have been subject to a longer experience of competitive tendering and service commissioning than the NHS [28], and tend to have a more structured approach for community engagement and user-involvement embedded in their organisational culture [29]. As a result, there appears to have been a shift in how public health commissioning is performed, from a more specialist-led investment approach to a more ‘business’-orientated approach adopted by many local councils, using best value frameworks.

In the new environment, there seems to be more opportunity for variation across the country in what activity is commissioned, and in who provides it (as well as how, where and to whom). The Localism Agenda [9] gives councils more freedom to innovate, to both drive down costs and meet local needs [30]. Considerable discretion was afforded to individual councils to interpret the full and detailed scope of their new functions and services [31]. This was important, given the independence of councils as democratic organisations, but it means that public health decision-making is now less amenable to central government control.

In the absence of strong central control, it is important to question the extent to which local problems can be solved locally without risking geographical inequity of services which underpin basic human rights [32, 33]. For the next couple of years, the annual public health budget devolved to local government in England will be around £3.3 billion (reducing by an average of 3.9% every year in real terms until 2020) [34]. Prior to the reforms, this budget would have been spent by PCTs, who were accountable for that spend to the DoH, via regional NHS authorities (now abolished) who were mainly concerned with overall NHS expenditure and financial sustainability of NHS healthcare services. Following the reforms, whilst the public health outcomes framework gives a clear sense of outcomes the DoH expects to see, the accountability for spending money is much weaker. Beyond a basic report to the Department on how the budget has been spent, there is very little role for formal state-driven accountability. In a way most uncharacteristic of the NHS, PHE has emphasised that it is there to support local councils, not performance manage them. Instead, there is a reliance on sector-led improvement, whereby councils review and support each other’s performance [35]. In addition, public health commissioning is coming under much closer scrutiny from elected members within the local council. Our research supported the idea that we can expect to see increasing variation in services, but it is far from clear what impact this will have on variations in outcomes.

Public health officers moving from the NHS to local councils have sometimes struggled to adjust to this different relationship with central government. From the point of view of commissioners, the lack of guidance and clarity from Government was often found to be unhelpful. In particular, public health officers expressed the need for more timely information, for instance, regarding responsibilities for commissioning across the fragmented system, or how the in-year budget cuts would be implemented [36]. In the absence of detailed information, public health teams were sometimes forced to make commissioning decisions based more on expediency than on need. In the new system, the DoH is defined as the ‘system leader’, improving people’s health and wellbeing through its stewardship of the public health system [37]. The concept of health stewardship implies a broad over-arching responsibility over the functioning of the system as a whole and, ultimately, over the health of the population [38]. However, we suggest that central government in England has yet to resolve some important stewardship issues, particularly around its role in securing resources, balancing competing interests and demands, and assuring delivery in the context of localism and the move of public health into local government. Moreover, there was little in our research to suggest that PHE have sufficient capacity, or have yet developed the strong relationships required, to provide meaningful support to local partners in the delivery of their vision.

Public health officers have also had to adjust to different roles and relationships relative to other actors at local level. Directors of public health were previously key decision makers on the executive boards of PCTs. Whilst they were often the first to be pushed back if cuts were required or budgets exceeded, DsPH had clear authority with regards to public health prioritisation. Following the reforms, they are expert advisers to elected members. Leadership for public health is more dispersed; decision-making is now more complex, and arguably subject to greater political ideology and personal interest. There may also be unforeseen consequences arising from the outcomes-based scrutiny of complex public health interventions, and from increased insecurity within the provider landscape. Many public health interventions require a long time-frame in which to bring about significant population health improvements. This doesn’t sit well with the short-termism of contemporary politics. As local councils struggle to cope with tighter budgets, public health officers may find it harder to convince their elected members of the added value of some of the public health services they commission.

Our research has highlighted the huge amount of change occurring in the commissioning (and decommissioning) of health improvement services in England. Whilst it will be important for the wider health system that key public health services are protected and improved (for instance, in smoking cessation, weight management and sexual health services), the public health specialists will need to capitalise on bringing about positive change through closer integration with the strategy and activity of the council. Commissioning for health improvement requires commissioners to focus on the modifiable determinants of health, taking a pro-active approach to improving individuals’ life chances and reducing social inequalities, rather than waiting until people are already ill and commissioning reactively. Local councils, due to their wider scope and responsibilities, are better placed than the NHS with its largely clinical orientation, to address a broad range of determinants, such as lifestyles, community, local economy and activities [30]. Our research, like the many case studies highlighted in a range of Local Government Association reports [39–42] has identified a range of positive examples where stronger and more direct public health involvement and influence across councils has brought about new opportunities. In their new ‘home’, and with the right support from their council, public health officers can be afforded the freedom to approach public health challenges in new ways. Local councils are also more adept than NHS organisations at broader level consultation and community engagement, which might afford new opportunities in line with the Ottawa Charter recommendations for public participation and empowerment [43].

The NHS continues to have a vital part to play in population health improvement, and the reforms hoped to bring about improved synergies between public health, NHS and social care. However, with public health moving ‘arms-length’ to the NHS, both health services commissioners and providers are becoming more remote to the local public health systems. Moreover, the vital co-ordination role of HWBs is not always working well locally [10]. Some health improvement services could, as a result, end up being disconnected from each other and from wider support. Similarly, services that are crucial to the achievement of health service objectives (such as reducing premature mortality from the major causes of death), but which are commissioned or provided by the council (e.g. weight management, smoking cessation and alcohol services), are at risk of being cut or changed. Our research has highlighted that there is much change in the way public health commissioning is done, who is doing it, and what is commissioned. Time (and further research) will tell if these changes are to result in improved outcomes and reduced inequalities. However, until there is a strong sense of shared ownership across local systems, and ‘whole system’ commissioning at local level, any opportunities afforded by the reforms to the public health system might be outweighed by the challenges of fragmentation and budget cuts.

## Conclusions

We found that the system created by the reforms was confused, continually changing, and - from the point of view of commissioning - more fragmented than before. In some ways, the move of public health into councils has brought about some of the opportunities associated with decentralisation – in particular, allowing for increased flexibility. However, most public health commissioners were essentially moved from one local organisation (NHS), to another (council), so the comparisons with decentralisation are limited. In this new local environment, former public health capacity has been at the same time freed and stifled. Public health commissioning is being more strongly influenced by a new set of decision-makers in the form of democratically elected councillors, with their own local knowledge, ideologies, and experiences. Meanwhile, many councils are bringing a more business-oriented approach to bear on public health commissioning, with greater scrutiny of outcomes in relation to spend. This is challenging the public health specialists and provider organisations, and changing the shape of health improvement services. Whilst we can expect to see increasing change and variation in services across England, it is far from clear what impact this will have on outcomes and on variations in outcomes.

## Abbreviations

CCG: Clinical Commissioning Group
DoH: Department of Health
DsPH: Directors of Public Health
HWB: Health and Wellbeing Board
NHS: National Health Service
PCT: Primary care trust
PHE: Public Health England

## Footnotes

1 The Duty of Best Value makes clear that councils should consider overall value – including social value – when considering service provision. Under the general Duty of Best Value, local authorities should “make arrangements to secure continuous improvement in the way in which its functions are exercised, having regard to a combination of economy, efficiency and effectiveness” https://www.gov.uk/government/publications/best-value-statutory-guidance--4.

## References

[1] Department of Health. Local government’s new public health functions. 2011. https://www.gov.uk/government/uploads/system/uploads/attachment_data/file/216710/dh_131905.pdf. Accessed 18 July 2016.

[2] Department of Health. New focus for public health - the health and social care act 2012. Factsheet B4. https://www.gov.uk/government/uploads/system/uploads/attachment_data/file/138263/B4.-Factsheet-New-focus-for-public-health-250412.pdf. Accessed 18 July 2016.

[3] Department of Health (2010). Healthy lives, healthy people: our strategy for public health in England.

[4] Rechel B, McKee M (2014). Facets of public health in Europe. European Observatory on Health Systems and Policy Series.

[5] World Health Organisation. Health 2020: The European policy for health and well-being. 2016. http://www.euro.who.int/en/health-topics/health-policy/health-2020-the-european-policy-for-health-and-well-being. Accessed 18 July 2016.

[6] Marks L, Cave S, Hunter D, Mason J, Peckham S, Wallace A (2011). Governance for health and wellbeing in the English NHS. J Health Serv Res Policy.

[7] Department of Health (2010). Equity and excellence: Liberating the NHS (cm 7881).

[8] Marks L, Cave S, Hunter DJ (2010). Public health governance: views of key stakeholders. Public Health.

[9] Department for Communities and Local Government (2011). A plain English guide to the Localism Act.

[10] Coleman A, Checkland K, Segar J, McDermott I, Harrison S, Peckham S (2014). Joining it up? Health and Wellbeing Boards in English Local Governance: Evidence from clinical commissioning groups and shadow health and wellbeing boards. Local Gov Stud.

[11] UK Parliament. Public health post-2013 inquiry launch. 2015. http://www.parliament.uk/business/committees/committees-a-z/commons-select/health-committee/news-parliament-20151/public-health-post-2013-inquiry-15-16/. Accessed 18 July 2016.

[12] Gadsby EW, Peckham S, Coleman A, et al. PHOENIX: Public health and obesity in England - the new infrastructure examined. First interim report: The scoping review. 2014. http://blogs.lshtm.ac.uk/prucomm/2016/07/15/phoenix-public-health-and-obesity-in-england-the-new-infrastructure-examined-2/. Accessed 18 July 2016.

[13] Riches N, Coleman A, Gadsby EW, Peckham S. The role of local authorities in health issues: A policy document analysis. London: PRUComm: Policy Research Unit on Commissioning and the Healthcare System; 2014. https://www.kent.ac.uk/chss/docs/CLG-report.pdf. Accessed 18 July 2016.

[14] Turnbull S (2002). Bricolage as an alternative approach to human resource development theory building. Hum Resour Dev Rev.

[15] Bryant C, Jary D (1991). Giddens’s theory of structuration: a critical approach.

[16] The Centre for Local Economic Strategies. Austerity uncovered: Final report prepared by the centre for local economic strategies, presented to TUC. December 2014. https://www.tuc.org.uk/sites/default/files/TUC%20Final%20Report%20Dec'14_1.pdf. Accessed 18 July 2016.

[17] Department of Health (2013). Developing a specification for lifestyle weight management services. Best practice guidance for tier 2 services.

[18] NICE (2014). Obesity: Identification, assessment and management. Guidelines CG189.

[19] Public Health England (2015). National mapping of weight management services. Provision of tier 2 and tier 3 services in England.

[20] Hughes C (2015). The rewards and challenges of setting up a tier 3 adult weight management service in primary care. Br J Obes.

[21] Stange KC (2009). The problem of fragmentation and the need for integrative solutions. Ann Fam Med.

[22] Pavolini E, Guillén A, editors. Health care systems in Europe under austerity: Institutional reforms and performance. UK: Palgrave Macmillan; 2013.

[23] Gorsky M, Lock K, Hogarth S (2014). Public health and England local government: historical perspectives on the impact of ‘returning home’. J Public Health.

[24] Mosca I (2006). Is decentralisation the real solution? A three country study. Health Policy.

[25] Robalino DA, Picazo OF, Voetberg A. Does fiscal decentralization improve health outcomes? - evidence from a cross-country analysis. Policy, Research working paper; no. WPS 2565. Washington, DC: World Bank; 2001. http://documents.worldbank.org/curated/en/832091468739534469/Does-fiscal-decentralization-improve-health-outcomes-evidence-from-a-cross-country-analysis.

[26] Peckham S, Exworthy M, Powell M, Greener I. Decentralisation, centralisation and devolution in publicly funded health services: Decentralisation as an organisational model for health care in England. Report for the national co-ordinating centre for NHS service delivery and organisation R & D (NCCSDO). UK: NCCSDO; 2005.

[27] Dowling B, Sheaff R, Pickard S (2008). Governance structures and accountability in primary care. Public Money Manage.

[28] Bovaird T (2016). The ins and outs of outsourcing and insourcing: What have we learnt from the past 30 years?. Public Money Manage.

[29] Schehrer S, Sexton S (2010). Involving users in commissioning local services.

[30] Royal Society for Public Health (2014). The RSPH guide to commissioning for health improvement.

[31] Peckham S, Gadsby EW, Coleman A, et al. PHOENIX: Public health and obesity in England - the new infrastructure examined. Final report. 2016. http://blogs.lshtm.ac.uk/prucomm/2016/07/15/phoenix-public-health-and-obesity-in-england-the-new-infrastructure-examined-2/. Accessed 18 July 2016.

[32] Pugh M (2014). Centralism versus localism? Democracy versus efficiency? The perennial challenges of Scottish local government organisation. History and Policy.

[33] Collins C, Green A (1994). Decentralization and primary health care: Some negative implications in developing countries. Int J Health Serv.

[34] Public health grants to be cut by £160m over next two years. Public Finance. 12 Feb 2016. http://www.publicfinance.co.uk/news/2016/02/public-health-grants-be-cut-ps160m-over-next-two-years. Accessed 18 July 2016.

[35] Buck D. Why we need to strengthen local authorities’ accountability for public health outcomes. The King’s Fund. 30 January 2013. http://www.kingsfund.org.uk/blog/2013/01/why-we-need-strengthen-local-authorities-accountability-public-health-outcomes. Accessed 18 July 2016.

[36] Department of Health (2015). Local authority public health grant allocations 2015/16. Government response to public consultation on in-year savings and equality and health inequality analysis.

[37] Department of Health. How is the Department of Health changing? 2013. http://webarchive.nationalarchives.gov.uk/20130805112926/http://healthandcare.dh.gov.uk/dh-change/. Accessed 18 July 2016.

[38] World Health Organisation (2000). The World Health Report 2000. Health systems: improving performance.

[39] Local Government Association, Public Health England (2014). Public health transformation nine months on: bedding in and reaching out.

[40] Local Government Association (2015). Public health transformation twenty months on: Adding value to tackle local health needs.

[41] Local Government Association (2015). Sexual health commissioning in local government.

[42] Local Government Association (2016). Healthy weight, healthy futures: Local government action to tackle childhood obesity.

[43] World Health Organisation (1986). The Ottawa charter for health promotion: First international conference on health promotion, Ottawa, 21 November.

